# Linguistic Creativity and Formal Thought Disorder in Schizophrenia

**DOI:** 10.1192/bjo.2024.137

**Published:** 2024-08-01

**Authors:** Louise Robinson, Dawn Archer, Alex Bartha, Gerasimos Chatzidamianos, Oliver Delgaram-Nejad

**Affiliations:** 1 Manchester Metropolitan University, Manchester, United Kingdom; 2 Amercian College of Greece, Greece; 3 East London NHS Foundation Trust, East London, United Kingdom; 4 Lancashire and South Cumbria NHS Foundation Trust, Lancashire, United Kingdom

## Abstract

**Aims:**

The present investigation was interested in whether formal thought disorder (FTD) in schizophrenia was in any way related to linguistic creativity. The project's main aims and research questions were the development of operational definitions of linguistic creativity and FTD in schizophrenia, an investigation of creative language processing in schizophrenia, and an investigation of creative language output in schizophrenia.

**Methods:**

We designed a psycholinguistic experiment and collected natural language data to build a specialised schizophrenia corpus. Recruitment for the psycholinguist experiment was challenged by the COVID pandemic and the technical abilities of clinical participants. Those data are thus underpowered and not reported in the results. We collected sufficient data for the construction of the specialised corpus.

**Results:**

We tested an operational definition of FTD in schizophrenia (the '4TD Framework') against our natural language dataset. There was good support for the framework, with grammatical and discourse tracking features reliably distinguishing clinical and comparison speakers (p < 0.05). We also examined concordance lines and grouped random concordances into error types. Error types were consistently similar across groups, suggesting that speech disturbances in schizophrenia are on a continuum with those of nonclinical speakers. We also conducted a keyness analysis to examine the key terms and semantic categories present in the corpus and noted significant differences in the clinical cohort. Clinical participants found discussion of the topic of linguistic creativity more challenging, deviating from topic more often. They also involved topics of emotional and personal concern at rates of up to 16 to 32 times more often than comparison participants in some cases.

**Conclusion:**

Our results provide support for the dysexecutive and dyssemantic hypotheses of FTD, as well as work on the Thought Language Index (TLI) that also suggests that language disturbances in schizophrenia and FTD are on a continuum with nonclinical speech. Further research is needed to understand how these phenomena are positioned in relation to FTD as a transdiagnostic entity.

